# Effect of diverting stoma for rectovaginal fistula: A protocol of systematic review and meta-analysis

**DOI:** 10.1097/MD.0000000000023202

**Published:** 2020-12-04

**Authors:** Wenqiang Fu, Sibin Yi, Mingwei An, Yong Tang, Luwei Tang, Yanru Wang, Yishun Yuan, Qiong Zhou, Yanfang Hu, Yiqi Wen

**Affiliations:** a Jiangxi University of Traditional Chinese Medicine; b The Affiliated Hospital of Jiangxi University of Traditional Chinese Medicine, Nanchang, P.R. China.

**Keywords:** colostomy, diversion, diverting colostomy, diverting stoma, metastatic stoma, ostomy, protocol, rectovaginal fistula, recto-vaginal fistula, stoma, systematic review

## Abstract

**Background:**

Rectovaginal fistula (RVF) is a pathologic channel between the anterior wall of the rectum and the posterior wall of the vagina, is rare, and the majority is of traumatic origin. The most common causes are obstetric trauma, local infection, rectal surgery or caused by chronic inflammatory bowel disease. Once the disease will seriously affect the patient's quality of life, and generally not self-healing, most require surgical intervention. At present, diverting stoma is mainly used in patients with severe RVF or complicated RVF or patients with Crohn disease. Due to the lack of large sample, linical studies, its clinical effectiveness is still controversial. The purpose of this systematic review is to evaluate the efficacy of diverting stoma in the treatment of diverting stoma.

**Methods:**

EMBASE, PubMed, the Cochrane Library, Chinese National Knowledge Infrastructure (CNKI), Wanfang Database, Chinese Biomedical Literature Database (CBM), and Chinese VIP Information will be searched systematically by 2 reviewers from the inception until October 2020. The original study that randomized controlled trials (RCTs), clinical controlled trials (CCTs), nonrandomized control trials (NCTs), and retrospective trials (RTs) of diverting stoma for RVF will be selected. In addition, similar searches will be conducted for the reference lists, researches in progress, and the citation lists of identified publications. Study selection, data extraction, and assessment of the quality will be performed independently by 2 reviewers who have been trained prior to data extraction. A meta-analysis will be conduct if the quantity and quality of the original studies included are satisfactory; otherwise, a descriptive analysis will be conducted. Review Manager 5.4 software (The Nordic Cochrane Centre, The Cochrane Collaboration, Copenhagen, Denmark) will be using for data synthesis and assessment the risk of bias according by Cochrane Handbook.

**Result:**

This study will provide a comprehensive review of current evidence for the treatment of diverting stoma on RVF.

**Conclusion:**

The conclusion of this study will provide a judging basis that whether the treatment of RVF with diverting stoma is effective.

**Inplasy registration number:**

INPLASY2020090070.

## 1 Introduction

Rectovaginal fistulas (RVFs) are defined as an abnormal epithelium connections between the anterior wall of the rectum and the posterior wall of the vagina. It represents approximately 5% of all anorectal fistulas by the report.^[1]^ Although uncommon, they cause great psychosocial burden to the patient because the symptoms are socially disabling. In addition, RVFs can lead to recurrent infections of the vagina or lower urinary tract.

In terms of etiology, various types of RVFs are distinguished. Congenital RVF is often associated with anorectal malformation and often requires anal reconstruction. Acquired RVF is often secondary to birth injury. Other etiologies include perianal sepsis, trauma, Crohn disease or are iatrogenic, etc. In older publications, obstetric fistulas are reported to represent 88% of RVFs, rendering them the most common type.^[2]^ In addition to obstetric fistulas, the use of staplers represents a risk factor for the development of RVFs secondary to rectal surgery with or without pouch creation. An important risk factor appears to be the use of staplers, especially if the so called double stapling technique is applied.^[3–5]^ Fistulas are primarily described in up to 10% of low anastomoses.^[3,6]^

At present, RVFs have no accepted classification. Most classifications are based on etiology, localization, and size. In the selection of surgical methods, it makes sense to distinguish between low and high RVFs. Low fistulas are those that can be reconstructed via an anal, perineal, or vaginal access, while high fistulas require an abdominal approach. Fistulas in the central third are very rare due to the location and the characteristics of the vaginal wall.

Spontaneous healing of a fistula is rare and the treatment for RVF includes conservative treatment and surgical repair. At present, the main surgical treatment include fistula excision, advanced flap, plugs, muscle interposition, however the success rate of surgical treatment varies from 0% to 80%.^[7–11]^ The majority of patients undergo more than one surgical operation. Although there are many surgical methods, none can be used to treat all types of RVF.

Although no relevant studies are currently available, diversion stoma is widely used in in clinical treatment for patients with RVF. For diversion stoma, in theory, helps control symptoms and supports fistula healing, this is consistent with the research findings of Corte et al^[12]^ they observed that temporary transversal stoma significantly increased the success rate of repair in 79 patients with RVF after 286 surgical procedures. Whereas in 2016, Lambertz et al^[13]^ found through retrospective study that the metastatic stoma did not help to improve the recurrence rate after RVF repair. At present, due to the lack of large sample clinical studies, the clinical effectiveness of diversion stoma is still controversial. Therefore, the purpose of this study was to summarize the original research on the treatment of RVFs with diversion stoma, so as to evaluate whether the treatment of RVFs with diversion stoma is really effective.

## 2 Methods

### 2.1 Registration

This protocol will be reported according to the Preferred Reporting Items for Systematic Reviews and Meta-analyses Protocols (PRISMA-P).^[14]^ It is registered in the INPLASY (registration number, INPLASY202090070; https://inplasy.com/inplasy-2020-9-0070/).

### 2.2 Inclusion criteria for this overview

PICOS will be applied, including Population, Intervention, Comparison, Outcome, and Study.

#### 2.2.1 Types of studies

Randomized controlled trials (RCTs), clinical controlled trials (CCTs), nonrandomized control trials (NCTs), and retrospective trials (RTs) with diversion stoma as the primary intervention for RVFs will be included, and other studies such as case reports and reviews will be excluded. No restrictions on country but language will be limited on English and Chinese.

#### 2.2.2 Types of participants

Participants diagnosed as RVFs will be included. No restrictions on age, race, etc.

#### 2.2.3 Types of interventions

Without limits on course and dose, we will include studies in which diversion stoma is the primary intervention and, if necessary, we will include studies in which diversion stoma is combined with other active treatments versus active treatment alone.

#### 2.2.4 Types of comparisons

The selected studies should testify that the interventions were compared with a control group composed of non-stoma (e.g., vaginal, coloanal or colorectal anastomosis, plug, seton drainage, and rectal advancement flap) or other active therapies.

#### 2.2.5 Outcomes

Primary outcome: The cure rate, recurrence rate, infection rates.

Secondary outcomes: Effective rate, inefficiency rate, adverse reactions, the operation time, length of hospital stay.

### 2.3 Search methods for study identification

#### 2.3.1 Electronic searches

Two researchers will retrieve the relevant trials in the following databases: EMBASE, PubMed, the Cochrane Library, Chinese National Knowledge Infrastructure (CNKI), Wanfang Database, Chinese Biomedical Literature Database (CBM), and Chinese VIP Information, from inception until October 2020 without restriction to languages and publication. A comprehensive retrieve strategy will be conducted, various combinations of MeSH items and free words will be searched synchronously, including “rectovaginal fistula,” “recto-vaginal fistula,” “diverting stoma,” “diverting colostomy,” and “metastatic stoma,” etc. The preliminary search strategy for PubMed is presented in Table 1.

**Table 1:** Search strategy (PubMed).

Order	Strategy
#1	Search “rectovaginal fistula”[Mesh] Sort by: Publication Date
#2	Search ((((rectovaginal fistula [Title/Abstract]) OR recto-vaginal fistula [Title/Abstract]) OR vaginal fistula [Title/Abstract]) OR rectofistula [Title/Abstract] Sort by: Publication Date
#3	#1 OR #2
#4	Search (((((((randomized controlled trial[Publication Type]) OR clinical trials [Publication Type]) OR Retrospective trials[Title/Abstract]) OR non-stoma[MeSH Subheading]) OR plug[Title/Abstract]) OR anastomosis[Title/Abstract]) OR seton drainage[Title/Abstract]) OR rectal advancement flap[Title/Abstract] Sort by: Publication Date
#5	Search (humans[MeSH Terms]) NOT animals[MeSH Terms] Sort by: Publication Date
#6	#4 AND #5
#7	Search (((diverting stoma[Title/Abstract]) OR metastatic stoma[Title/Abstract]) OR ostomy[Title/Abstract]) Sort by: Publication Date
#8	#3 AND #6 AND #7

#### 2.3.2 Searching other resources

The relevant published references and citation list will be retrieved in Web of Science. In addition, the relevant systematic reviews or overview will also be identified for additional relevant studies. Moreover, relevant paper versions of medical journals and journals will be screened to ensure that the original studies that not included in the electronic databases could be included possibly.

### 2.4 Data collection and analysis

#### 2.4.1 Study selection

**Figure 1:**
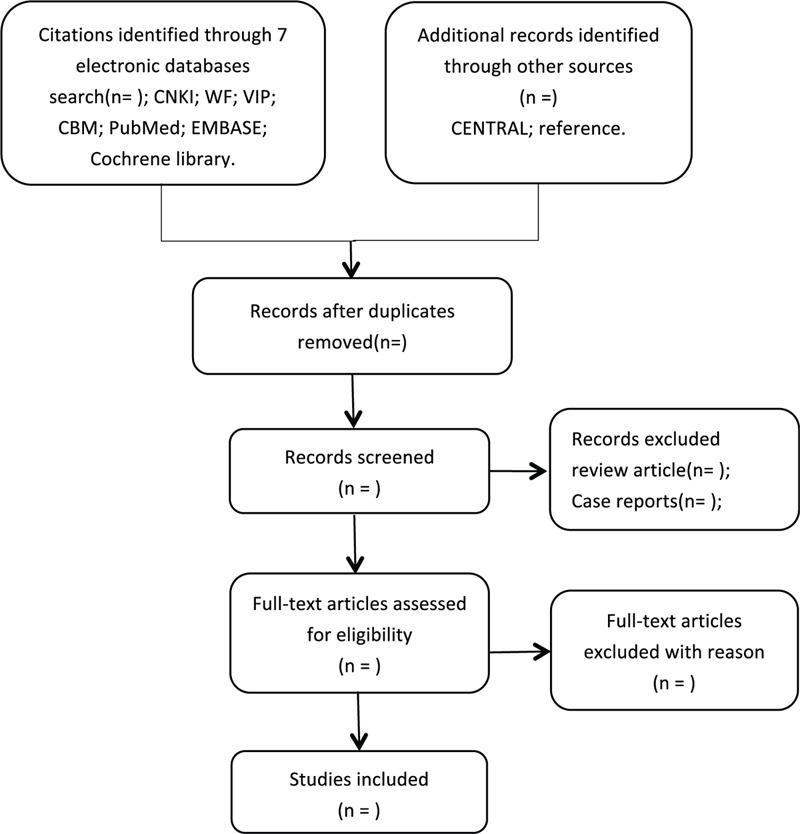
Flowchart of literature selection.

All reviewers undergo rigorous training prior to selecting the study. The initial screening was conducted independently by two highly trained researchers. After retrieval, duplicate studies will be removed by EndNote (X9). After deleting duplicate references, 2 independent reviewers (WQF and YT) will evaluate and filter the titles, abstracts, and keywords of the search study, according to established selection criteria, and then we will get the full text of all relevant studies. Excluded studies will be documented and interpreted in detail. Disagreements between 2 reviewers will be resolved through discussion. In case no agreement can be reached, a third researcher (MWA) will help resolve the disagreement, and the final opinion of the researcher shall prevail. Study selection will be performed in accordance with the PRISMA flowchart (Fig. 1).

#### 2.4.2 Data extraction and management

Before data extraction, a unified data extraction table will be designed, and data extraction will also be conducted by 2 reviewers independently (YRW and YSY). The proposed extracted information includes: General information: author, country, year of publication, study design, and database; Population characteristics: age, baseline diseases, and sample size; Methodological characteristics: information sources, intervention(s), comparison(s), bias assessment, etc. Any objections will be discussed by 2 reviewers, and further objections will be arbitrated by the third author (QZ).

#### 2.4.3 Assessment of risk of bias

To systematically evaluate the quality of each of the studies that final included. Two reviewers (YFH and YQW) will assess the risk of bias for each included study according to the Cochrane handbook. It will be divided into 3 levels eventually (“high risk of bias,” “medium risk of bias,” and “low risk of bias”).^[15]^ The specific evaluation items include the following 7 aspects: generation of random sequence, allocation concealment, blindness of participants and personnel, blindness of outcome assessment, incomplete outcome data, selective reporting, and other bias.

#### 2.4.4 Measures of treatment effect

Review Manager (RevMan V 5.4) (The Nordic Cochrane Centre) will be used for data analysis and quantitative data synthesis. We will use the weight mean difference (WMD) and 95% confidence interval (CI) to measure the continuous variables, while the results of dichotomous variables will using risk ratio (RR) and its 95% confidence interval (CI).

#### 2.4.5 Dealing with missing data

If the specific information we need to collect are not be reported, the reviewer (SBY) will attempt to contact the original author for relevant information by telephone or e-mail. If the required information is not available, it will be explained in the article. Then, the missing data will be assumed to be “missing at random (MAR)” and “missing not at random (MNAR)” according to the Cochrane Handbook.^[16]^ For the data MAR, the analysis will rely on existing data, while we will filling the missing data with replacement values and make a sensitivity analysis to examine the potential impact of missing information, if necessary.

#### 2.4.6 Assessment of heterogeneity

Heterogeneity refers to the difference between studies in the systematic review,^[14]^ and the value of *I*^2^ represents the heterogeneity after data synthesis. We will use *I*^2^ to assess statistical heterogeneity between trials. If the *I*^2^ < 50%, that indicates slight or no heterogeneity in the evidence of the combined results, while *I*^2^ ≥ 50%, it means studies with high heterogeneity. The fixed effects model will be adopted when the *P* > .1 and *I*^2^ < 50%, while apply the random effect if *P* < .1 and *I*^2^ ≥ 50%.

#### 2.4.7 Assessment of reporting bias

An assessment of the reported bias will be presented in the form of a funnel plot. If the points on both sides of the funnel plot are scattered and asymmetric, it is considered that there is a report bias and the reliability of this study is low. On the contrary, if the point distribution on both sides of the funnel plot is symmetrical, we believe that there is no or very low reporting bias, and the results of this study are reliable.

#### 2.4.8 Data synthesis and subgroup analysis

All analysis will be done through RevMan 5.4. According to heterogeneity assessment, MD or RR were calculated using fixed or random effects models. In addition, if the *I*^2^ obtained after data consolidation is >50% and the *P* value is <.1, sensitivity or subgroup analysis will be performed to exclude the source of heterogeneity. If the included original research data are insufficient for quantitative analysis, the review will only represent and summarize the evidence.

#### 2.4.9 Sensitivity analysis

If the results show significant heterogeneity and the number of included studies is sufficient, sensitivity analysis will be performed to identify the quality and robustness of the meta-analysis result, which includes assessing the impact of sample size, methodological elements and the characteristic of research and missing data.

#### 2.4.10 Grading the quality of evidence

The quality of evidence will be evaluated using the The Grading of Recommendations Assessment, Development and Evaluation (GRADE).^[17]^ The quality of evidences will be rated on 4 levels (high, medium, low, or very low). Two reviewers (YSY and YT) will conduct the assessment process separately and describe in detail the reasons for downgraded or upgraded outcomes affecting the quality of evidence to guarantee the reliability and transparency of results.

## 3 Discussion

Rectovaginal fistula is an abnormal passage between the rectum and the vagina. Although clinically rare, they do occur. Patients often complain of vaginal discharge or discharge of pus, which will seriously affect the patient's physiology, psychology, and even sexual function. At present, there is no recognized therapy that can be applied to every type of RVF, and the main therapeutic methods include conservative therapy, surgical therapy, and Interposition of biomaterials. Conservative treatment includes local irrigation, sitz bath, abscess drainage, and effective antibiotic application. It is mainly used in patients with RVF who are early caused by anorectal surgical injury and anastomotic infection, with fresh fistula, small diameter, and less inflammation. Due to the small scope of application, there have been few reports on the efficacy of conservative treatment in recent years. Surgical treatment mainly includes Endorectalclosure, Transcircuit closure, and Transperineal closure etc. However the success rate of surgical treatment varies from 0% to 80%.^[7–11]^ Interposition biomaterial includes ploughing in fibrin adhesive, fistula, plug, and biomembrane etc, and have also only been published in the form of case reports. Diversion stomata, as a clinically useful approach, in theory, helps control symptoms and supports conducing healing. Due to the lack of large sample clinical studies, its clinical effectiveness is still controversial. Lambertz's et al^[13]^ study in 2016 suggested that the metastatic stoma did not help to improve the recurrence rate after RVF repair. However, the study of Corte et al^[12]^ suggests that temporary diversion stoma can significantly improve the success rate of repair. To date, there is no reliable comprehensive review of the treatment of RVFs with diversion stoma. We conducted this study to assess the efficacy of diversion stoma in the treatment of RVFs and to provide clinical staff with a reliable treatment regimen. In addition, through this study, it is believed that more and higher quality original studies will be designed and carried out to provide more accurate guidance for the treatment of RVFs.

## Acknowledgments

The authors would like to thank the following people who either provided feedback on the protocol or supported the development of the methods: Wenqiang Fu, Sibin Yi, Mingwei An, Luwei Tang, Yanru Wang, Yishun Yuan, Qiong Zhou, Yanfang Hu, Yiqi Wen.

## Author contributions

**Conceptualization:** Wenqiang Fu, Mingwei An.

**Data curation:** Wenqiang Fu, Sibin Yi, Luwei Tang, Yishun Yuan, Qiong Zhou, Yanfang Hu.

**Formal analysis:** Wenqiang Fu, Sibin Yi.

**Funding acquisition:** Wenqiang Fu.

**Investigation:** Wenqiang Fu, Mingwei An.

**Methodology:** Wenqiang Fu, Yong Tang, Yanru Wang, Yiqi Wen.

**Software:** Sibin Yi, Luwei Tang.

**Supervision:** Mingwei An, Yanru Wang, Yishun Yuan.

**Writing – original draft:** Wenqiang Fu, Mingwei An, Sibin Yi, Yong Tang.

**Writing – review & editing:** Mingwei An, Luwei Tang, Qiong Zhou, Yanfang Hu.
